# Use of Baicalin-Conjugated Gold Nanoparticles for Apoptotic Induction of Breast Cancer Cells

**DOI:** 10.1186/s11671-016-1586-3

**Published:** 2016-08-30

**Authors:** Donghyun Lee, Wan-Kyu Ko, Deok-Sang Hwang, Dong Nyoung Heo, Sang Jin Lee, Min Heo, Kook-Sun Lee, Ji-Yoon Ahn, Junyoung Jo, Il Keun Kwon

**Affiliations:** 1 Department of Dentistry, Graduate School, Kyung Hee University, Seoul, 02477 Korea; 2 Department of Dental Materials, School of Dentistry, Kyung Hee University, Seoul, 02477 Korea; 3 Department of Korean Gynecology, Conmaul Hospital, Kyung Hee University, Seoul, 02477 Korea; 4 Department of Clinical Korean Medicine, Graduate School, Kyung Hee Univeristy, Seoul, 02477 Korea; 5 Department of Oral and Maxillofacial Radiology, School of Dentistry, Kyung Hee University, Seoul, 02477 Korea

**Keywords:** Baicalin, Gold nanoparticle, Beta cyclodextrin, Anti-cancer, Apoptosis

## Abstract

Baicalin (BC) has been used for cancer therapy due to its multiple effects as an anti-cancer drug. However, the effective delivery of this molecule to targeted cells is difficult. Gold nanoparticles (AuNPs) conjugated with thiolated beta cyclodextrin (AuNP-S-β-CD) were used as a delivery vector in this study. Cell viability tests were evaluated by cell counting kit-8 (CCK) and live/dead cell assay. To demonstrate the proliferation inhibition effect on Michigan Cancer Foundation-7 (MCF-7) cells by BC, we analyzed using Hoechst 33342 staining assay and gel electrophoresis. The S-β-CD conjugated AuNPs were characterized by transmission electron microscopy (TEM), 1H nuclear magnetic resonance (^1^H NMR), and ultraviolet visible (UV-vis) spectroscopy. AuNP-S-β-CD with approximately 40 μM of BC loaded by inclusion complex showed an inhibition effect on MCF-7 cells by inducing apoptosis. Apoptosis test results were evaluated by analyzing the expression of typical apoptic markers such as cleaved caspase-3, full length caspase-3, and apaf-1 in western blot assay. These results demonstrated that AuNP-S-β-CD-BC inhibited the proliferation of cancerous MCF-7 cells by inducing apoptosis. These findings suggested that AuNP-S-β-CD-BC could be a promising agent for chemotherapeutic usage.

## Background

Breast cancer is one of the most common forms of cancer in women in the world. The rate of breast cancer incidence is increasing rapidly because of changes in diet, breastfeeding methods, and the environment. Many kinds of therapy have been tried for treatment of breast cancer [1]. The most successful treatments have been chemotherapy and radiotherapy. However, these therapies are often accompanied by side-effects [2, 3].

In order to alleviate these adverse side-effects, studies are actively underway to develop naturally based treatments for breast cancer [4–7]. Scutellaria baicalensis Georgi (SB), a common medicinal herb, contains various flavonoids such as baicalin (BC), baicalein, wogonin, wogonoside, oroxylin A, and oroxylin A-7. These are well-known constituents which contribute to SB biological activity [8]. Especially, BC and baicalein have been reported for inhibition of cancer proliferation as well as inducing apoptosis of cancer cells [9–12]. BC has useful properties for use as a drug; however, it is difficult to deliver BC in a targeted fashion towards cancer cells.

Recently, in the biomaterials research field, gold nanoparticles (AuNPs) bearing moieties which target receptors on the cell surface have been used for delivery of materials into cells through endocytosis [13–16]. In addition, immune cells containing AuNPs were used to utilize in cancer therapy field [17–20]. According to Albanese and Chan, AuNPs have no cytotoxicity and have a great capacity for cellular uptake [21]. Furthermore, biological drugs such as proteins, DNA, and RNA have been investigated as materials that can be delivered via various sizes of AuNPs or gold nanorods [22–29].

In this study, we designed a functionalized AuNPs modified using thiolated beta-cyclodextrin (AuNP-S-β-CD) as a means to load BC. Cell counting and western blot assays were conducted to prove the anti-cancer effectiveness of AuNP-S-β-CD-BC. Michigan Cancer Foundation-7 (MCF-7) cells, which are typical breast cancer cells, were used in all the cellular tests.

## Methods

Gold (III) chloride hydrate (99.999 % trace metals basis), sodium citrate, iodine, beta cyclodextrin, sodium methoxide, baicalin, and Hoechst 33342 stain were purchased from Sigma-Aldrich (St. Louis, MO, USA). Dulbecco’s modified eagle medium (DMEM), fetal bovine serum (FBS), penicillin streptomycin (Pen strep), and Dulbecco’s phosphate-buffered saline (DPBS) were purchased from GIBCO BRL (Invitrogen Co., USA). Antibody for β-actin was purchased from Santa Cruz Biotechnology (Santa Cruz, CA, USA). Antibodies for cleaved caspase-3, full-length caspase-3, and apaf-1 were purchased from Cell Signaling Technology (Beverly, MA, USA).

MCF-7 cells were purchased from Invitrogen (Carlsbad, CA, USA) and cultured in a tissue culture plate filled with DMEM (containing 1 % PS and 10 % FBS) in a 37 °C heated-humidified atmosphere with 5 % CO_2_. MCF-7 cells were seeded into 24-well plates (1 × 10^5^ cells/well) and incubated for 24 and 48 h, respectively. At each predetermined timepoint, the optical densities of the cells were evaluated by using a cell counting kit-8 (CCK, Dojindo, Kumamoto, Japan). In order to quantify, we measured the cell viability percentage amongst all groups. The intensity was measured by a microplate reader (BioRad, USA) at a wavelength of 450 nm. The live/dead cell assay kit, including calcein-AM/ethidium homodimer-1 (EthD-1) was used to demonstrate the inhibition effect of BC qualitatively.

MCF-7 cells were seeded at a density of 2 × 10^5^ cells/well in a medium containing 100 μM of BC for Hoechst 33342 and DNA fragmentation assay. After 2 days, the cells were stained with Hoechst 33342 solution for 1 h in a 37 °C heated-humidified atmosphere with 5 % CO_2_, and were then observed by a fluorescent microscope (Olympus IX71, Japan). For DNA fragmentation assay, the cells were lysed by using a lysis buffer containing a protease inhibitor. A total of 250 μg of gDNA was extracted from each group using a gDNA purification kit (Taiwan). The gDNA was tested using gel electrophoresis in a 2 % agarose gel and measured by ChemiDoc XRS System (Bio-Rad, Hercules, CA, USA).

AuNPs were produced by reducing gold chloride hydrate as previously described [30]. Briefly, 0.02 % gold chloride hydrate solution (800 ml) was boiled to 100 °C under a reflux condenser, and 2 % sodium citrate solution (15 ml) was added into the gold chloride hydrate solution. The mixed solution reaction changed color to dark purple. The temperature was held for 15 min and then allowed to cool gradually to room temperature. S-β-CD was prepared as described in our previous study [31]; 50 μM of BC and 50 μM of AuNP-S-β-CD were mixed and ultra-sonicated for 5 min to load the BC into the β-CD. The loading volume of BC on AuNP-S-β-CD was calculated by comparing the optical density of the BC solution group with the AuNP-S-β-CD-BC solution group. The synthesized/conjugated materials were analyzed by transmission electron microscopy (TEM) (H-7100, Hitachi, Japan), 1H nuclear magnetic resonance spectrometer (^1^H NMR spectrometer, INOVA400, CA, USA), and ultraviolet visible (UV-vis) spectroscopy (1650PC spectrophotometer, Shimadzu, Japan).

MCF-7 cells were seeded at a density of 1 × 10^5^ cells/well with a medium-containing AuNP-S-β-CD and AuNP-S-β-CD-BC in 6-well plates and incubated for 24 and 48 h, respectively. At the predetermined hour, MCF-7 cells were lysed by the addition of cold RIPA lysis buffer containing 0.5 M Tris-HCl, pH 7.4, 1.5 M NaCl, 2.5 % deoxycholic acid, 10 % NP-40, and 10 mM EDTA along with protease and phosphatase inhibitors. The cell lysates were incubated in an ice box for 30 min and then centrifuged at 1.3 × 10^4^ rpm for 10 min. Aliquots of 40 μg of equal amounts of protein was added into sodium dodecyl sulfate and loaded into polyacrylamide gel for electrophoreses (SDS-PAGE). These were subsequently transferred using a nitrocellulose transfer membrane. After blocking the nitrocellulose transfer membrane with 5 % skim milk, the membrane was probed with cleaved caspase-3, full-length caspase-3, apaf-1, and β-actin followed by incubation with an appropriate secondary antibody conjugated to horseradish peroxidase. Signals were detected using a ChemiDoc XRS System (Bio-Rad), and β-actin was regarded as an internal control group.

Statistical analysis was performed using Student’s *t* test. All values were expressed as means ± standard deviations, and all the experimental groups compared with the control group.

## Results and Discussion

**Fig. 1:**
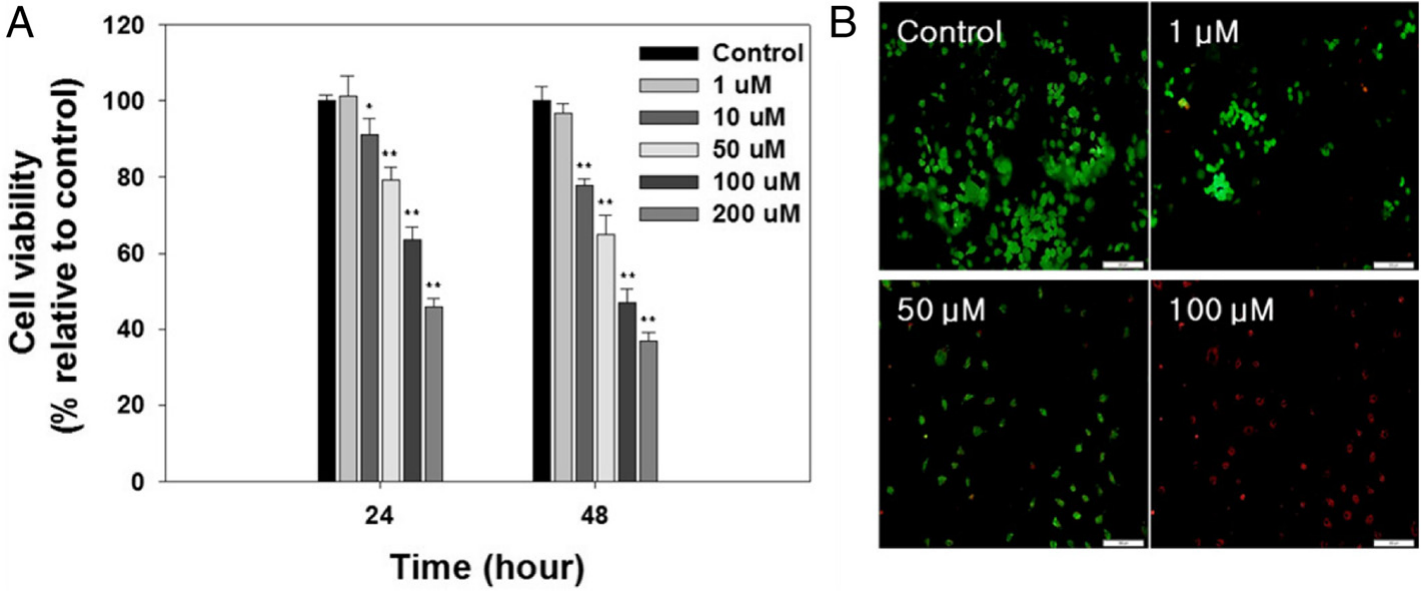
Evaluation of cell viability in MCF-7 cells using cell counting kit (**a**) and live and dead cell assay (**b**). *Single asterisk* indicates significant difference of *p* < 0.05, *double asterisks* indicate significant difference of *p* < 0.001 (*N* = 3)

We performed a cell viability test in order to confirm the anti-cancer effectiveness of BC. The proliferation inhibitory rate was evaluated using a microplate leader. Cells were treated with various concentrations of BC (1, 10, 50, 100, and 200 μM) and were measured at 24 and 48 h, respectively. After 24 h, the results of the cell viability test demonstrated cell viability at 101, 91, 79, 64, and 46 % for doses of 1, 10, 50, 100, and 200 μM BC, respectively (Fig. 1a). After 48 h of incubation, the viability of each group was further reduced as compared with the 24-h groups. The live/dead cell assay also showed that BC inhibited MCF-7 proliferation in a dose-dependent manner. Especially, almost all MCF-7 cells were dead when exposed to doses greater than 100 μM of BC (Fig. 1b).

**Fig. 2:**
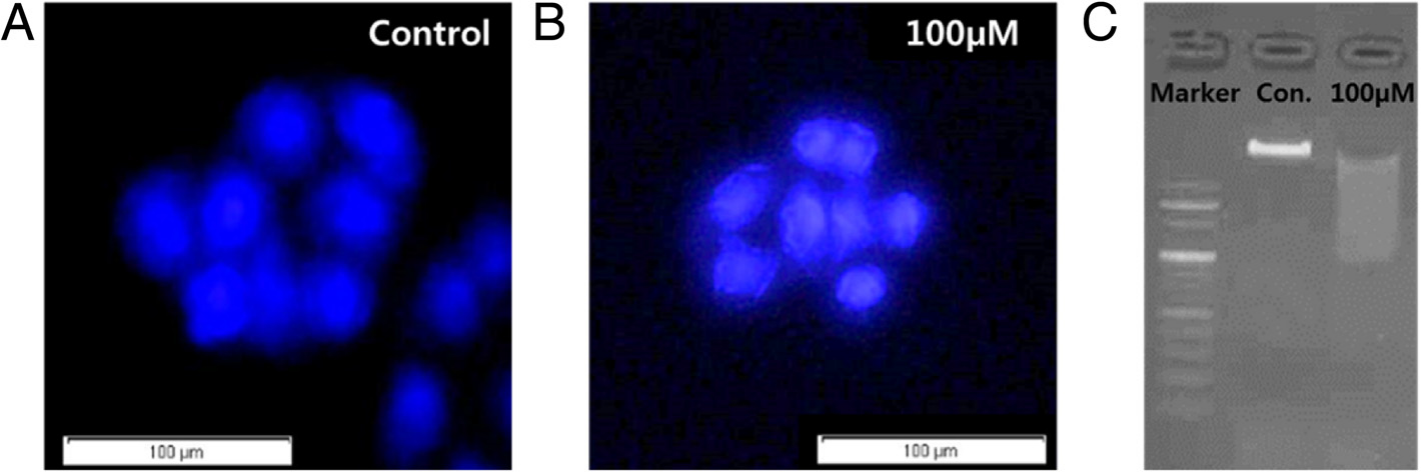
Fluorescent images of MCF-7 cells (**a, b**) under apoptosis. DNA fragmentation induced by BC as determined by gel electrophoresis (**c**)

Hoechst 33342 staining and DNA fragmentation assay were carried out to analyze the BC-induced apoptosis effect (Fig. 2). In the fluorescence microscopic image, 100-μM BC-treated cells showed significantly higher nuclear fragmentation which confirms apoptosis (Fig. 2b). Figure 2c shows the “ladder-shape” DNA fragmentation which also indicates DNA apoptosis [32]. These results show that apoptosis occurred in the BC-treated group.

**Fig. 3:**
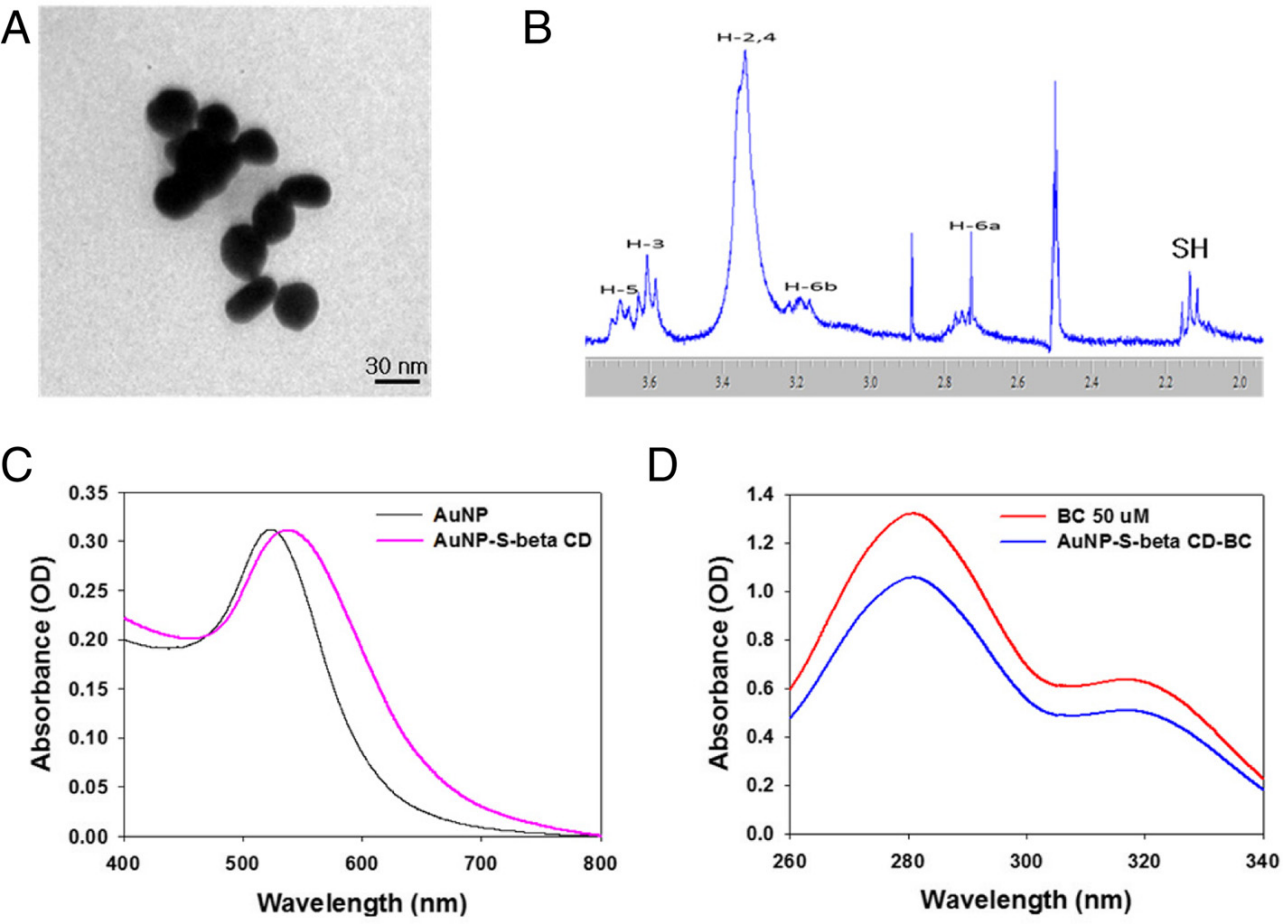
TEM image of gold nanoparticles (**a**), 1H NMR graph of S-β-CD (**b**), absorbances of AuNP and AuNP-S-β-CD (**c**), absorbance spectra of BC and AuNP-SH-β-CD-BC (**d**) as measured by UV-vis spectroscopy

We designed an AuNP-S-β-CD which can be a delivery vector for BC by serving as an inclusion complex. The conjugated complexes were fully characterized by TEM, ^1^H NMR, and UV-vis spectroscopy. In this study, we manufactured 30-nm-diameter AuNPs which have no inherent toxicity towards the body [33]. The AuNPs were conjugated with S-β-CD in order to provide for inclusion of BC. Figure 3a shows that the AuNPs had a spherical shape. The OH terminal groups of β-CD were substituted with a thiol group in order to allow for conjugation to the AuNPs. The substituted β-CD demonstrated SH peak in the ^1^H NMR graph (Fig. 3b). The thiol group of the β-CD enables bonding of β-CD with AuNP [34]. We confirmed S-β-CD conjugation to AuNPs by using UV-vis spectroscopy (Fig. 3c). The maximum absorbance of the control AuNPs group was found to occur at 525 nm. For the AuNP-S-β-CD group, the maximum absorbance shifted to the right (533 nm) as compared with the AuNPs group. This shift in the maximum absorbance wavelength indicates successful attachment of the S-β-CD on AuNPs [35]. We sonicated BC with AuNP-S-β-CD in order to generate the AuNP-S-β-CD-BC complex and confirmed that approximatively 40 μM BC was loaded onto the AuNP-S-β-CD (Fig. 3d).

**Fig. 4:**
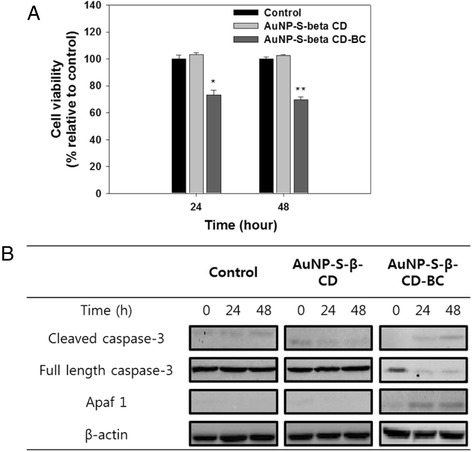
Anti-proliferation effects of AuNP-S-β-CD-BC against MCF-7 cells (**a**) the expression of cleaved caspase-3, full cleaved caspase-3, and Apaf-1 signals as measured from samples treated with AuNP-S-β-CD and AuNP-S-β-CD-BC (**b**). *Single asterisk* indicates significant difference of *p* < 0.05, *double asterisks* indicate significant difference of *p* < 0.001 (*N* = 3)

The AuNP-S-β-CD-BC group also demonstrated inhibitory effects against MCF-7 cells after 48 h of incubation (Fig. 4a). The proliferation of MCF-7 cells was significantly inhibited by the loaded BC released from the AuNP-S-β-CD. When apoptosis occurs in cancer cells, the apaf-1 signal is expressed. Subsequently, the full-length caspase-3 signal decreases and the cleaved caspase-3 signal increases [36]. The western blot assay results demonstrate that apoptosis occurs in AuNP-S-β-CD-BC group cells (Fig. 4b). Apaf-1 and cleaved caspase-3 bands from the AuNP-S-β-CD-BC groups were observed to have a higher intensity as compared with AuNP-S-β-CD alone and control group. These results demonstrate that the BC complexed onto AuNP-S-β-CD-induced apoptosis of MCF-7 cells.

## Conclusions

We prepared AuNP-S-β-CD-BC complex for delivery of BC as a chemotherapeutic strategy. Spherical AuNPs, 30 nm in diameter, were successfully conjugated with S-β-CD. Subsequently, BC was complexed onto these AuNP-S-β-CD. The anti-proliferation effectiveness of the AuNP-S-β-CD system complexed with BC was confirmed using MCF-7 cells. All the results indicated that BC from this system could induce apoptosis of MCF-7 cells. Although future studies will be necessary to confirm anti-cancer effects on the in vivo animal studies, these findings and previous studies reporting the functionalized gold nanoparticle for drug delivery system suggest that the baicalin loaded AuNPs are effective materials for anti-cancer treatment.

## Abbreviations

1H NMR: 1H nuclear magnetic resonance
AuNPs: Gold nanoparticles
AuNP-S-β-CD: AuNPs conjugated with thiolated beta cyclodextrin
BC: Baicalin
CCK: Cell counting kit-8
MCF-7: Michigan cancer foundation-7
SB: Scutellaria baicalensis georgi
SDS-PAGE: Sodium dodecyl sulfate and loaded into polyacrylamide gel for electrophoreses
S-β-CD: Thiolated beta cyclodextrin
TEM: Transmission electron microscopy
UV-vis: Ultraviolet visible
β-CD: Beta cyclodextrin
